# flowDiv: a new pipeline for analyzing flow cytometric diversity

**DOI:** 10.1186/s12859-019-2787-4

**Published:** 2019-05-28

**Authors:** Bruno M. S. Wanderley, Daniel S. A. Araújo, María V. Quiroga, André M. Amado, Adrião D. D. Neto, Hugo Sarmento, Sebastián D. Metz, Fernando Unrein

**Affiliations:** 1 Instituto Metrópole Digital, Universidade Federal do Rio Grande do Norte, Natal, Brazil; 2 Departamento de Oceanografia e Limnologia, Universidade Federal do Rio Grande do Norte, Natal, Brazil; 3 Instituto Tecnológico de Chascomús (INTECH), Universidad Nacional de San Martín (UNSAM) - Consejo Nacional de Investigaciones Científicas y Técnicas (CONICET), Buenos Aires, Argentina; 4 Departamento de Biologia, Universidade Federal de Juiz de Fora, Juiz de Fora, Brazil; 5 Departamento de Hidrobiologia, Universidade Federal de São Carlos, São Carlos, Brazil

**Keywords:** Flow cytometry, Cytometric diversity, R language

## Abstract

**Background:**

Flow cytometry (FCM) is one of the most commonly used technologies for analysis of numerous biological systems at the cellular level, from cancer cells to microbial communities. Its high potential and wide applicability led to the development of various analytical protocols, which are often not interchangeable between fields of expertise. Environmental science in particular faces difficulty in adapting to non-specific protocols, mainly because of the highly heterogeneous nature of environmental samples. This variety, although it is intrinsic to environmental studies, makes it difficult to adjust analytical protocols to maintain both mathematical formalism and comprehensible biological interpretations, principally for questions that rely on the evaluation of differences between cytograms, an approach also termed cytometric diversity. Despite the availability of promising bioinformatic tools conceived for or adapted to cytometric diversity, most of them still cannot deal with common technical issues such as the integration of differently acquired datasets, the optimal number of bins, and the effective correlation of bins to previously known cytometric populations.

**Results:**

To address these and other questions, we have developed flowDiv, an R language pipeline for analysis of environmental flow cytometry data. Here, we present the rationale for flowDiv and apply the method to a real dataset from 31 freshwater lakes in Patagonia, Argentina, to reveal significant aspects of their cytometric diversities.

**Conclusions:**

flowDiv provides a rather intuitive way of proceeding with FCM analysis, as it combines formal mathematical solutions and biological rationales in an intuitive framework specifically designed to explore cytometric diversity.

**Electronic supplementary material:**

The online version of this article (10.1186/s12859-019-2787-4) contains supplementary material, which is available to authorized users.

## Background

Flow cytometry (FCM) is a highly versatile technology that has been widely applied in various fields, from industrial processes to medical and environmental research [1–3]. One of the greatest appeals of FCM stems from its rapid and reliable assessment of detailed information on single or multiple cells from any given cell population. This versatility has led to its rapid adoption in different areas of expertise, resulting in a wide range of applications and the development of various specialized protocols for data analysis, which are usually not interchangeable.

Environmental sciences in particular face difficulty in adapting non-specific protocols to their context, mainly because of the highly heterogeneous nature of environmental samples [4, 5]. However, this heterogeneity is central to environmental studies, as it reveals much about the properties of any given community, for instance microbial communities [4, 5]. Precisely for this reason, the environmental FCM community has been directing efforts to developing methods focused on the depiction of this heterogeneity through cytograms, a concept presently explored under the closely related names of “cytometric pattern” [6], “cytometric fingerprint” [6] and “cytometric diversity” [7, 8].

Studies of cytometric resemblance have made great efforts with respect to their implementation [9–12] and their critical assessment [6], but the most suitable methods to manipulate environmental data are still under debate. In one sense, reasonable choices would favor methods that appropriately balance mathematical formalism and comprehensible biological interpretations, in a very similar manner to those that are extensively applied in the field of ecology [13].

Notably, most available tools in some sense do incorporate ecological rationales into their methods, but the possibility of explicitly applying them to describe cytometric resemblances remains underexploited. Indeed, since this approach was pioneered more than 20 years ago by Li (1997) under the term “cytometric diversity” [7], only a few studies have delved into this line [8, 14–16].

Briefly, Li’s seminal approach consists of binning cytograms and converting them to contingency tables of events, counting them by applying 16 ×16 Cartesian grids to each two-dimensional cytogram. Each contingency table summarizes a pool of non-taxonomic units, the bins, which are then used to derive some measures of biodiversity. Notwithstanding its astounding implications, some important aspects of the method were left incomplete in the original method, namely: *i*) the issue of low dimensionality; *ii*) the optimal number of bins; *iii*) the integration of differently acquired datasets; *iv*) pairwise resemblances; and *v*) bin’s explicit roles on cytometric diversity.

The issue of low dimensionality refers to the difficulty of dealing with more than two channels at a time. Although this suffices in many situations [14], selection of only two channels impedes deeper scrutiny of the information, since it does not allow efficient control of the additional features of the data at hand, notably for multicolor assays.

The optimal number of bins relates to a formal rather than empirical definition of the appropriate number of bins prior to the data analysis. While the most parsimonious solution at this point is to narrow the bin width to limits in which the largest amount of information data is preserved while still allowing less-intensive computation, this issue still lacks a closed-form solution.

Integration of differently acquired datasets encompasses the idea that a proper comparison between cytograms requires them to be set to common perspectives in order to correctly match the bins of interest. This is a highly restrictive constraint that requires all files to be acquired strictly within the same protocol guidelines. To some extent, however, such a constraint could theoretically be relaxed if some sort of perspective guides, such as internal standards (e.g., latex beads), could be used for a perspective control of cytograms, as is usually done in traditional FCM analysis. This solution, although promising, has not yet been explored.

Last are the issues regarding two closely linked aspects, easily deducible from but not covered in the first implementation of the method: pairwise resemblances and the bins’ explicit roles in cytometric diversity.

Pairwise resemblances derive from the fact that because individual cytograms can be depicted by their individual properties, clearly it should be possible to infer their pairwise (dis)similarities as well. The diversity indices (*α* indices) described in the original work concern only the particular features of a system. Hence, if the *α* diversities of two or more cytograms can be inferred, their resemblances, a concept referred to in ecology as *β* diversity, can also be assessed.

Measuring the cytometric *β* diversity, on the other hand, intuitively raises questions regarding the bins’ contributions to the differences detected, notably how the bin properties, such as position and number of counts, could lead to differences between cytograms, and in what way these properties effectively correlate with previously known cytometric populations. This is fundamental information, without which diversity measures provide only limited information [17].

In this article, we suggest solutions for these fundamental questions by discussing the implementation of flowDiv, a pipeline for analyzing environmental flow cytometry data, devised as an extended full implementation of Li’s ideas. To illustrate the potential of flowDiv, we applied it to reveal important aspects of the cytometric diversity from 31 lakes in Argentine Patagonia.

## Design and implementation

**Fig. 1:**
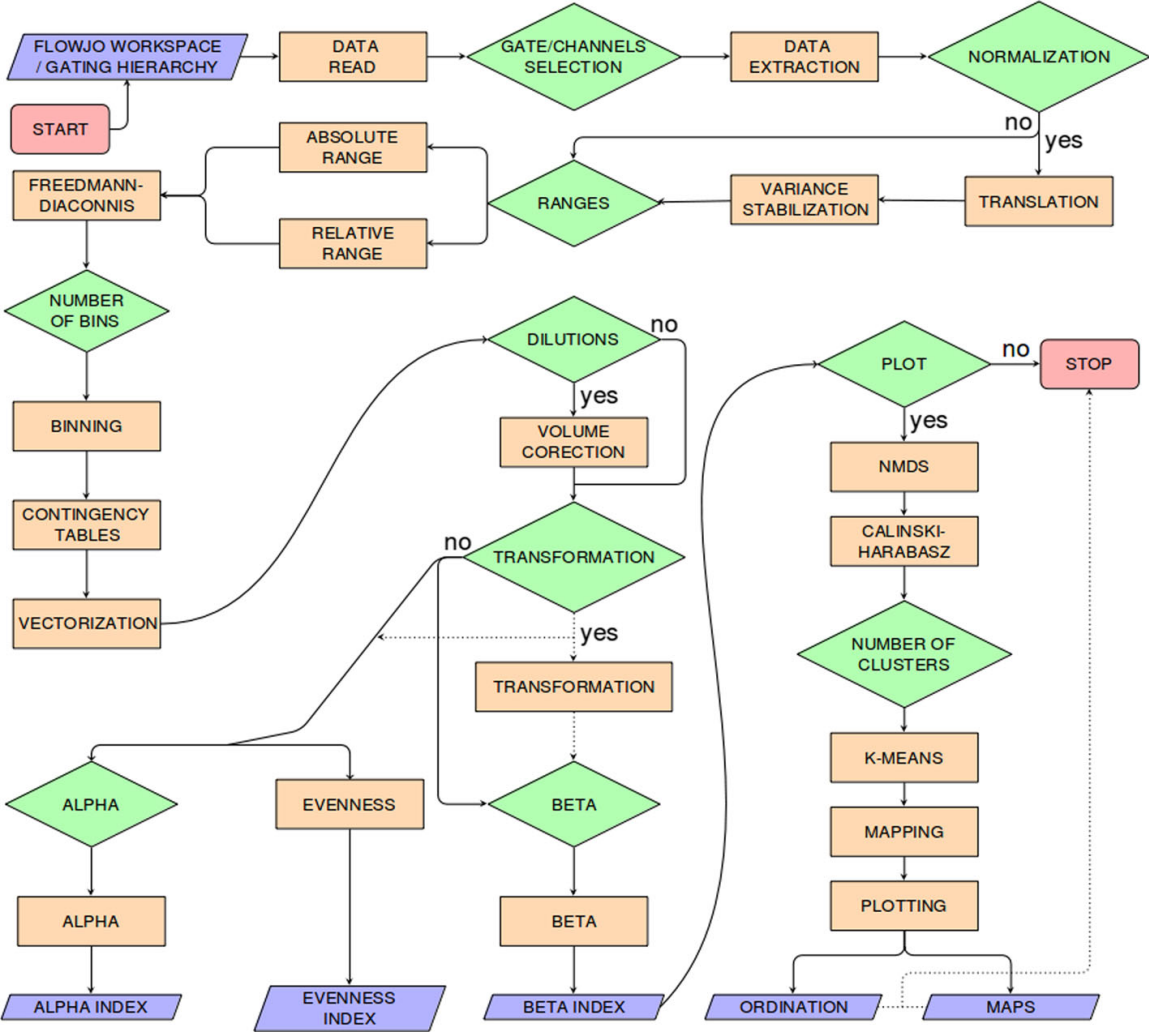
Schematic view of the flowDiv workflow

flowDiv is implemented in the R language and is structured in 19 stages of processing and 11 stages of oriented decision (Fig. 1). Here we describe the rationale behind each stage in detail.

### Data read

The first step of the pipeline consists of reading and parsing preprocessed (i.e. compensated, normalized or transformed) [18] FCS data. Input may be structured either as FlowJo^®^ workspaces or, equivalently, as GatingSet R objects.

This process is a wrapper for some flowWorkspace [19] and flowCore [20] subroutines. It is intended to reduce the complexity of the overall analysis by reducing the number of required software programs to two at most. This allows a manageable and more reproducible execution of the assay.

### Gate selection

Once imported, the next action consists of the extraction of user-defined regions of interest, the gates.

Gates are regions defined by their channels and respective borders (limits) that must be provided to the algorithm. While borders are internally and automatically parsed, information about which channels to use must be defined empirically by the analyst.

This is one of the key steps of the algorithm, as it expands the data analysis to higher dimensions, allowing more than two channels to be set per analysis.

### Range definitions

For any selected channel, a histogram is generated with equal numbers of bins. First, the channel ranges and bin width must be outlined.

The ranges within which channels will be binned can be defined either by the relative maximum and minimum values of the pooled set of channels (dynamic ranges), or by setting absolute limits for each channel separately (fixed ranges).

Fixed ranges define static limits for the histograms, producing a global model for comparative analyses between different runs of the algorithm. Dynamic ranges, on the other hand, mean that only the limits spanned by the data are considered in the binning process, maximizing the information gain in the analysis.

### Normalization

To fit specific scenarios where the data include any control standards (e.g., beads) but are acquired under different protocol guidelines – namely for scenarios where the operator accounts for changes in the data while controlling for the variance – we provide an approach to set the data to a common perspective through a translational transformation of the data (termed, in our pipeline, normalization).

Formally, in each vector *v*=(*a*_1_,*a*_2_,...,*a*_*n*_), representing the channels features of a particular cytogram, we apply a transformation *T*, such as:

1 $$ T(v) = \left(a_{1}+\triangle b_{1}, a_{2}+\triangle b_{2},\ldots,a_{n}+\triangle b_{n}\right)   $$

Where *b*=(△*b*_1_,△*b*_2_,…,△*b*_*n*_) represents the displacement coordinates for each point. Here, *b* is the vector of the difference computed between the mean bead values of each channel and a grand mean, calculated from the pooled mean bead values for each channel of all cytograms in the set, such as:

2 $$ \triangle b_{ij} = \frac{\sum_{1}^{j} \overline{w}_{ij}}{n}-\overline{w}_{ij}  $$

Where $\overline {w}_{ij}$ is the representation of the arithmetic mean of bead values from channel *i* of cytogram *j*, and *n* corresponds to the absolute number of samples (cytograms).

Following translation, flowDiv runs a variance stabilization of the data based on the approach implemented by Azada et al. (2015) in the flowVS package [21].Briefly, these steps proceed to an inverse hyperbolic sine (asinh) transformation of data with the form:

3 $$ T(v_{i}) = asinh (v_{i}/c_{i})   $$

Where *c*_*i*_ equals a normalization factor, calculated for each channel *i* individually [21].

### Binning

After the ranges are defined and the data centralized, the algorithm proceeds to data binning: here, the analyst will be asked how many bins should be used in the histogram construction.

In view of the innate high variability of natural environments, it is not reasonable to define a basic number of bins that represent any kind of data. Binning should be changeable, according to the nature of the data at hand. To deal with this, we have implemented a subroutine for inferring the optimum number of bins, which is based on the Freedman-Diaconis rule [22]:

4 $$ bins_{ij} = \left\lceil {\frac{max(x_{ij}) - min(x_{ij})}{2 \cdot IQR(x_{ij}) \cdot n_{j}^{\frac{-1}{3}}} } \right\rceil  $$

Where *bins*_*ij*_ represents the ceiling number of bins for channel *i* of sample *j*; *n* is the number of observations for the sample *j*; *IQR* stands for interquartile range and *x*_*ij*_ is the channel vector *i* of sample *j*.

The optimum number of bins, *bins*_*b*_, is calculated simply from the arithmetic mean of all suggested bins pooled, as follows:

5 $$ bins_{b} = \frac{\sum_{1}^{i} \sum_{1}^{j} bins_{ij}}{max(i)\cdot max(j)}   $$

### Contingency tables

The binning process results in the creation of common, mutually exclusive, exhaustive and ordered classes (bins), which are then cross-tabulated and used to construct an *n*-dimensional contingency table *S* in the form:

6 $$ S = \{ x_{i_{k}} \mid i = 1,2,\ldots, m \quad \text{and} \quad k=1,2,\ldots,n \}  $$

Where $x_{i_{k}}$ corresponds to the number of counts for bin *i* of channel *k*.

### Vectorization

Each *n*-dimensional contingency table is further linearly transformed to column vectors, in a process known as vectorization, creating a one-to-one correspondence between elements of the multidimensional space and elements of its transformed form, as follows:

7 $$ V_{j}=vec(S_{j}) = \{ x_{1_{1}},\ldots,x_{1_{2}},\ldots,x_{i_{k}} \}  $$

The rationale behind this step is to make the data more manageable for subsequent manipulation, by reducing the data dimensionality while keeping the information unchanged.

### Volume correction

In some circumstances, environmental samples are previously diluted before running a flow cytometer experiment: such dilutions may occur as a direct consequence of stain, fixative or beads addition, or as a requirement to keep event counting within a protocol-specified range [2].

All of these situations must be appropriately considered in the final calculations, in order to correctly determine the real frequency of any targeted event. In our pipeline, we deal with dilution bias by applying a user-defined correction factor to each individual sample, such as:

8 $$ F = W \cdot D_{cf}  $$

Where *W* is an *n*x*j* matrix composed of all column vectors *V*_*j*_, and *D*_*cf*_ is a diagonal matrix in which element *d*_*ij*_ corresponds to the ratio between the minimum true volume passed (i.e., the real volume analyzed, considered after correcting for dilutions of any nature) of all samples pooled and the true volume passed for sample *j*. The minimum value is chosen to downweight any background noise generated in relatively long runs.

### Diversity analysis

After vectorization, each cytogram is further used to derive three measures of biological diversity: *α*-diversity, species evenness, and *β*-diversity.

To make these steps as feasible and adjustable as possible, we take advantage of another important suite of tools available in the vegan package [23] to provide a wide range of *α* and *β* indices for calculation. By incorporating vegan::diversity() and vegan::betadiver() functions in its workflow, flowDiv allows analysts to manage, in addition to one evenness index (Pielou’s index), three different indices of *α* diversity (Shannon-Weaver, Simpson and inverse Simpson) and 24 indices of *β* diversity, as reviewed by Koleff et al. (2003)[24].

#### Nestedness and turnover

Some of the available *β* indices have particularly useful properties for FCM data analysis, as is the case for Bray-Curtis [25] semimetrics. Besides being an appropriate index for raw count data, it can also be partitioned into two very informative complementary components, nestedness and turnover.

In an abstract sense, nestedness and turnover correspond, respectively, to AND and XOR relationships between two sets of bins (e.g., Baselga, 2009 [26]). In the present context, these two components serve as convenient proxies to detail how the differences in cytograms might be partitioned between bin superposition (nestedness) or bin differential counting (turnover).

Because of their clear utility, both indices are also incorporated in our pipeline, as a wrapper of the betapart:bray.part() function, and are automatically called when the Bray-Curtis dissimilarity is chosen.

### Transformations

To accommodate other ecologically meaningful distance measures (see [27] and [23] for details), we have also incorporated another optional step, transformation. Internally, this process is simply a wrapper for the decostand{vegan} function.

### Ordination analysis, clusterization and mapping

Once *β*-diversity indices are acquired, the next step consists of an ordination and biplot of the results (cytograms and bins) to help in further investigations of the contributions of bins to the observed differences. Since Non-Metric Multidimensional Scaling (nMDS) has the convenient property of accommodating any (dis)similarity measure handled by flowDiv [28], we applied this technique in our pipeline.

For the purpose of keeping track of broader regions of the contingency tables while allowing further inspection of plots using traditional visual approaches, flowDiv proceeds to the clusterization of the bin ordination scores to generate a single masking image, which is further applied onto each cytogram individually. This step provides a novel and straightforward way of visually interpreting the bin ordination directly in cytograms.

For clusterization, we use the *K*-means clustering method. Briefly, the goal of *K*-means clustering is to partition *n* observations into *k* mutually exclusive clusters. More formally, *K*-means aims to minimize a squared error function *J*, such as:

9 $$ \underset{c}{\arg\min} \ J =\underset{c}{\arg\min} \ \sum_{i=1}^{k} \sum_{j=1}^{n} \lVert x_{ji} - \mu_{i} \rVert_{2}^{2}   $$

Where ∥*x*_*ij*_−*μ*_*i*_∥_2_ is the Euclidean distance between a data point *x*_*j*_, belonging to cluster *i*, and the cluster center *μ*_*i*_. In the flowDiv context, the set of observations *x*=(*x*_1_,*x*_2_,...,*x*_*n*_) represents the set of 2-dimensional real vectors, defined by each of the *n* bin ordination scores obtained in the previous step.

#### Choice of *K*

Determining the ideal number of clusters, *K*, is not a trivial task unless analysts can make some reasonable practical assumptions about the optimum number of clusters. For other situations, a data-driven process should be used, and considering these explicitly, we adopted the Calinski-Harabasz [29] criterion to guide our definition of the best number of clusters. The Calinski-Harabasz criterion, *C*, is defined as:

10 $$ C = \frac{n-K}{K-1} \cdot \frac{BG_{SS}}{WG_{SS}}   $$

In the formula, *n* is the number of bins, *K* is the number of clusters, *WG*_*SS*_ is the sum of squares within the clusters, and *BG*_*SS*_ is the sum of squares between the clusters.

flowDiv tests *K* iteratively within a pragmatically defined range, from one to ten clusters, and the lowest *C* is set as a suggestion of the appropriate number of clusters.

## Example of use

### Introduction

To evaluate flowDiv, we analyzed bacterioplankton data from 31 lakes in Patagonia, Argentina, collected in the provinces of Chubut, Santa Cruz and Tierra del Fuego. These aquatic systems seem to be an appropriate benchmark for our pipeline, as they have a clear geospatial gradient as well as a multitude of different ecological characteristics that have already been shown to be reflected in their bacterial community structure [30–32].

To assess the flowDiv consistency, we also briefly contrasted it with five other available cytometric fingerprint computation tools: Dalmatian Plot [11], Cytometric Histogram Image Comparison (CHIC) [10], Cytometric Barcoding (CyBar) [12], FlowFP [9] and PhenoFlow [16].

### Material and methods

#### Datasets

This case study focused on three different datasets for each aquatic system: (1) 12 morphometric, physical, and chemical environmental variables; (2) flow cytometry FCS files, manually gated for bacterioplankton populations; and (3) bacterial polymerase chain reaction denaturing gradient gel electrophoresis (PCR-DGGE) bands’ relative intensities. Detailed information about the study sites, protocols, sampling design and environmental parameters was provided by Schiaffino et al. [30–32].

#### Environmental parameters

Samples were collected from the euphotic zone, during spring in the years 2007 (Chubut and Santa Cruz) and 2008 (Tierra del Fuego) along a latitudinal gradient from 45^∘^55’S to 54^∘^36’S. The following parameters were recorded: latitude, longitude, area, temperature, pH, electrical conductivity, dissolved oxygen (DO), dissolved nitrogen (DN), diffuse attenuation coefficient (*K*_*d*_), chlorophyll a (Chla), phosphate, and dissolved organic carbon (DOC).

#### Flow cytometry data

Flow cytometry data were acquired with a FACSCalibur (Becton Dickinson) flow cytometer equipped with a standard 15 mW blue argon-ion (488 nm emission) laser and a red laser diode (635 nm), using 1 *μ* fluorescent beads as i nternal controls and SYTO 13 as the nucleic-acid stain. Bacterioplankton populations were manually gated by their cytometric signature in detection channels for 90^∘^ light scatter (bacterial cell size and structural complexity), green fluorescence (nucleic acid content), and red fluorescence (fluorescence spillover from the dye SYTO 13), following guidelines by Gasol et al. 2015 [2]. The gating strategy was performed with FlowJo ^®^ v.10 software.

#### flowDiv settings

The cytogram ranges were dynamically defined and were binned through channels SSC-H (90^∘^ light scatter), FL1-H (green fluorescence), and FL3-H (red fluorescence) for 75 bins per channel. Shannon diversity, richness, Pielou’s evenness, and Bray-Curtis semimetrics, as well as the components nestedness and turnover were evaluated. Bin ordination scores were clustered into five groups as suggested by the Calinski-Harabasz criterion.

#### Statistics

All statistics were performed with R version 3.3.2 (2016), using the following additional packages: vegan [23], RVAideMemoire [33], gvlma [34], corrplot [35], gplots [36] and ggplot2 [37].

Principal components analysis (PCA), non-metric multidimensional scaling (NMDS), and regression of environmental vectors onto ordination plots were based on the stats::prcomp(), vegan::metaMDS() and vegan::envfit() functions.

Tests on ordination score centroids were conducted with permutational multivariate analysis of variance (PERMANOVA) while controlling for spatial variation. PERMANOVA and tests for multivariate homoscedasticity were done with vegan::adonis() and vegan::betadisper() respectively.

Linear models were conducted after checking for model assumptions by gvlma::gvlma(). Additionally, to correct for unbalanced factors in the models, we merged mesotrophic (n = 13) and eutrophic (n = 4) groups (cf. Schiaffino et al. (2013)[31]) into a single class, termed “meso-eutrophic”.

Distance matrices for pairwise comparisons and Mantel’s test were run with vegan::vegdist() and vegan::mantel(). All tests were performed assuming an *α* level equal to 0.05.

Details of the coding for statistical analysis, including the datasets generated and analyzed, can be found online at https://github.com/bmsw/Supplementary-Code/blob/master/Statistical_Analysis.R.

### Results and discussion

#### Alpha diversity and evenness

**Fig. 2:**
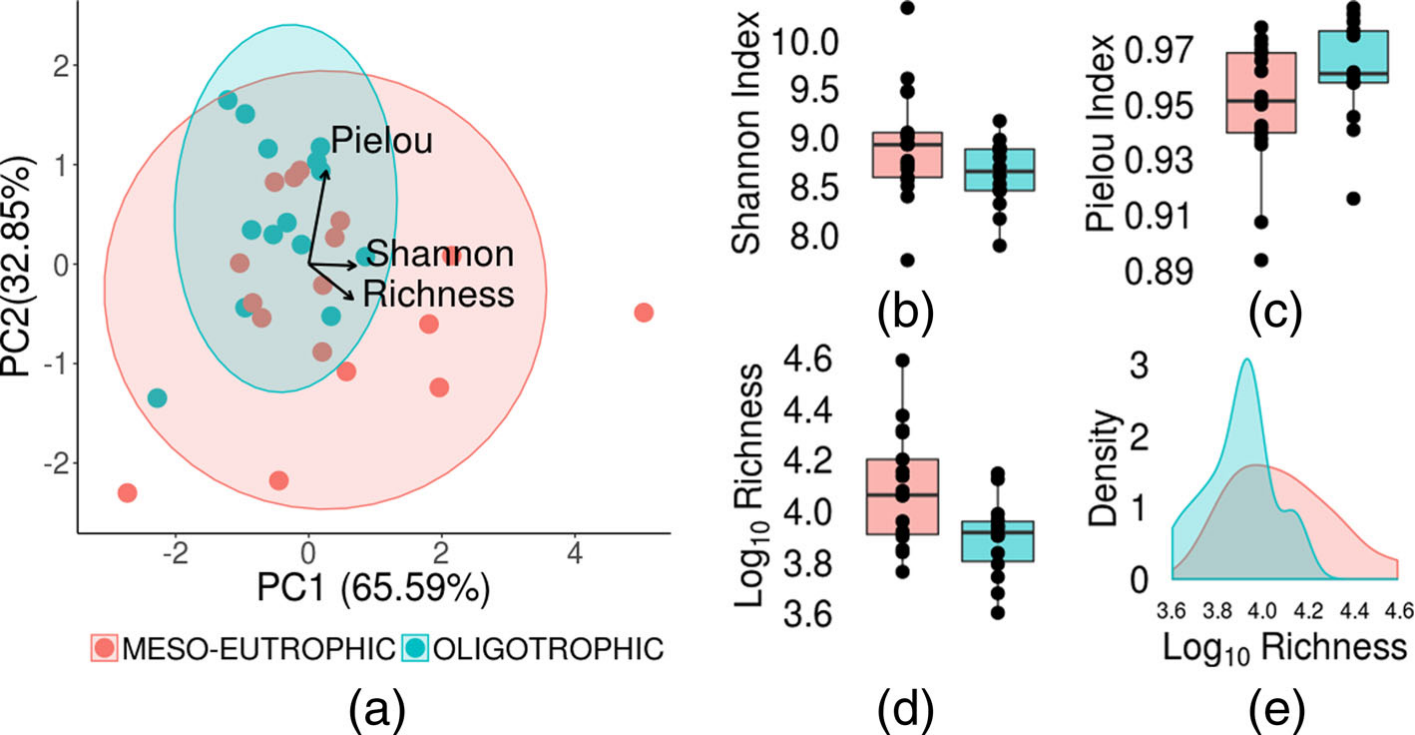
PCA correlation biplot **a**, boxplots **b**, **c** and **d** and density plot **e** computed from 31 Patagonian lakes using cytometric richness, Pielou’s evenness, and the Shannon index. Shaded areas in the PCA biplot represent 95% confidence ellipses

Principal components analysis (PCA) of cytometric indices revealed a smoothed separation pattern among the samples (Fig. 2a), suggesting that differences among waterbody trophic states could be associated with cytometric diversity, richness in particular. To test this hypothesis, we performed a Wilcoxon rank sum test under the null hypothesis that average cytometric richness is not dependent on the trophic status of a waterbody. The null hypothesis, however, was not supported (*P*<0.05).

**Fig. 3:**
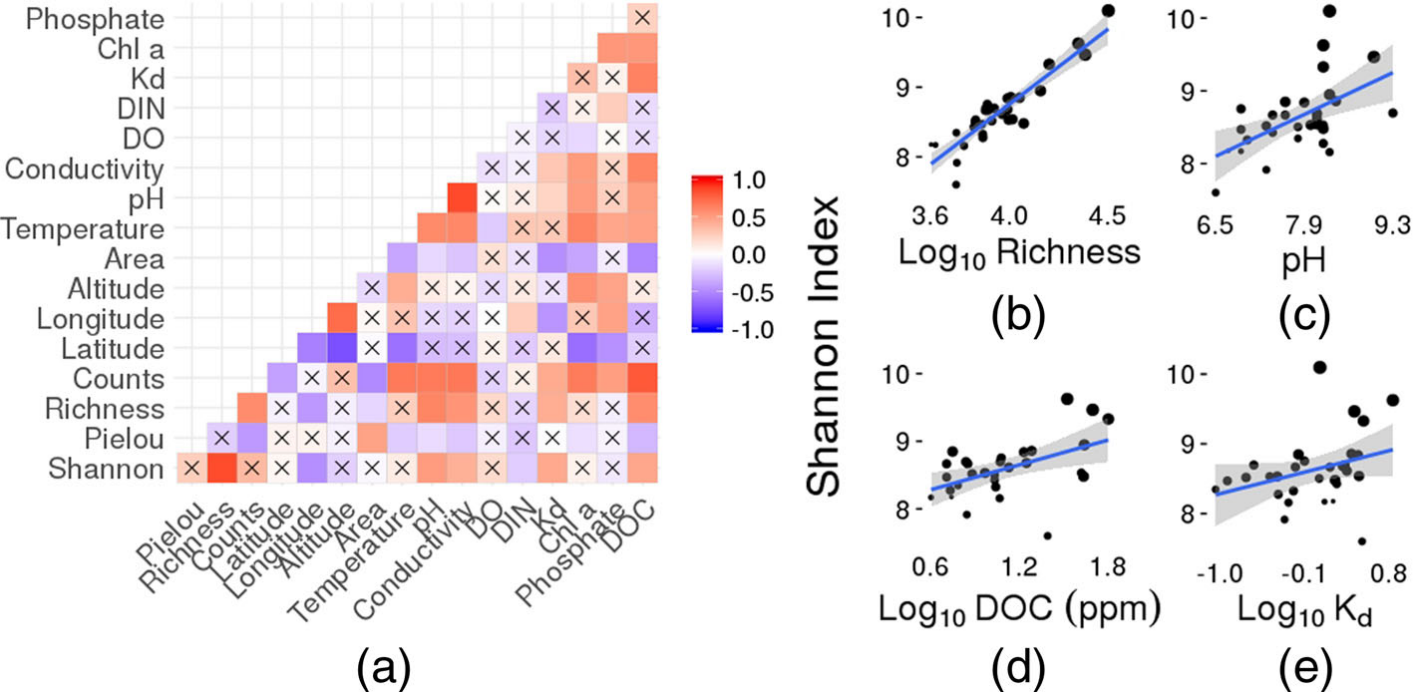
Correlation matrix based on Spearman’s rank correlation coefficient **a** of cytometric indices and environmental variables. Black crosses indicate non-significant correlations. Linear regression models of the Shannon-Weaver index and *Log*_10_ cytometric richness **b**, pH **c**, *Log*_10_DOC **d** and *Log*_10_*K*_*d*_**e**. Point sizes reflect *Log*_10_ cytometric richness values

Spearman’s rank correlation, in turn, showed that eight of 13 environmental variables showed significant relationships to the cytometric indices (Fig. 3).

We note that pH, Kd and DOC are variables directly associated with the trophic status. It has been demonstrated that at low DOC concentrations, only some bacterial specialists are able to actively incorporate the various types of organic matter effectively [38], and as a consequence, the bacterial diversity would be low. Accordingly, the positive relationship observed between *α* diversity and DOC is in line with the idea that higher concentrations of DOC, which are associated with a more-diverse DOC composition, would result in higher diversity of the bacteria that use these varieties of compounds.

#### Beta diversity

**Fig. 4:**
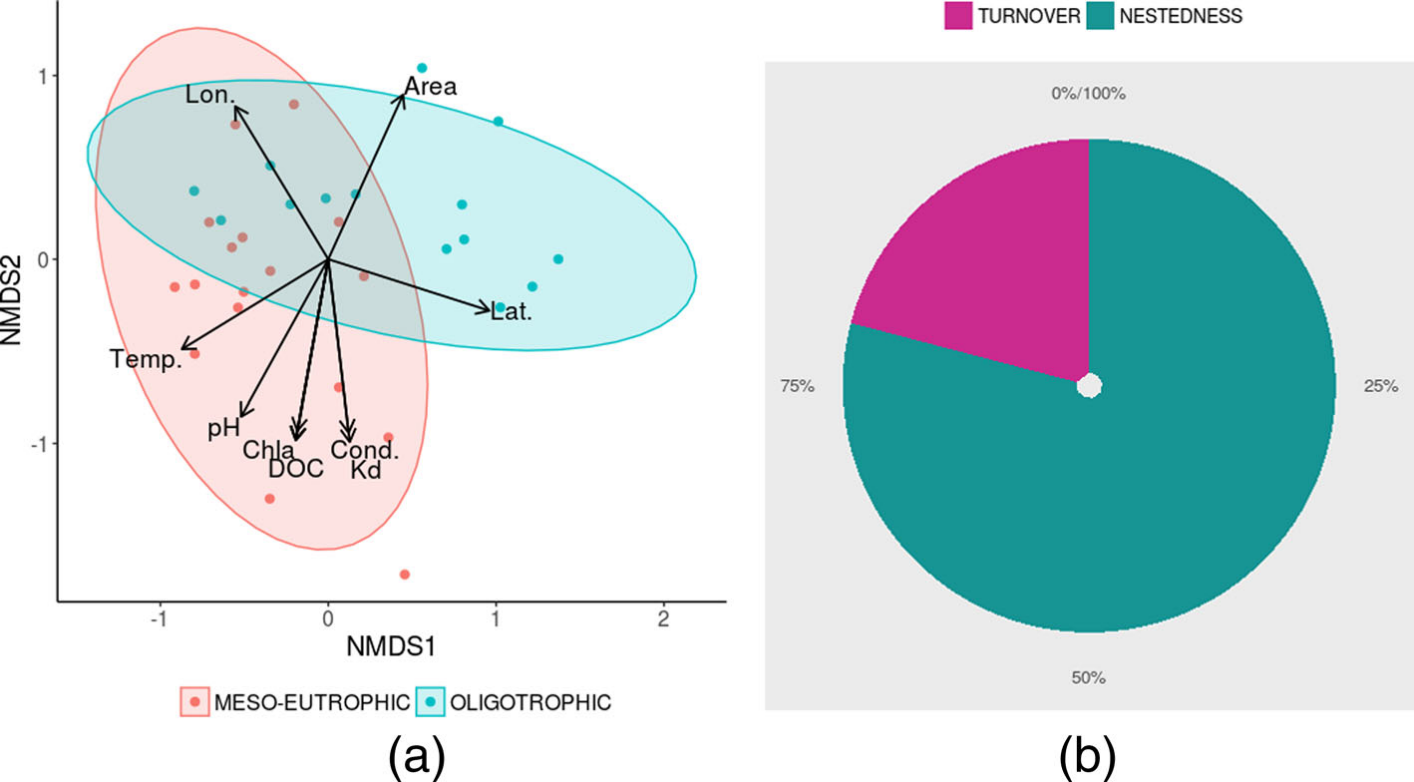
**a** NMDS of 31 Patagonian lakes computed in Bray-Curtis distance (Stress = 0.10) jointly plotted with fitted significant variables: dissolved organic carbon (DOC), chlorophyll a (Chla), pH, Kd, latitude (Lat), longitude (Lon), area, altitude, and temperature (Temp.); **b** Pie chart of partitioned Bray-Curtis distance (nestedness and turnover). Shaded areas in the NMDS plot represent 95% confidence ellipses

Ordination of Bray-Curtis distances indicated apparent differences in group means (Fig 4a), which were later confirmed by the PERMANOVA test (P <0.05). The ordination scores, in turn, showed significant linear correlations with nine environmental variables: DOC, chlorophyll a, pH, Kd, latitude, longitude, area, altitude, and temperature (Fig. 4a).

Furthermore, distance partitioning revealed that nestedness accounted for the major differences among the systems (Fig. 4b).

#### Ordination analysis, clusterization and mapping

**Fig. 5:**
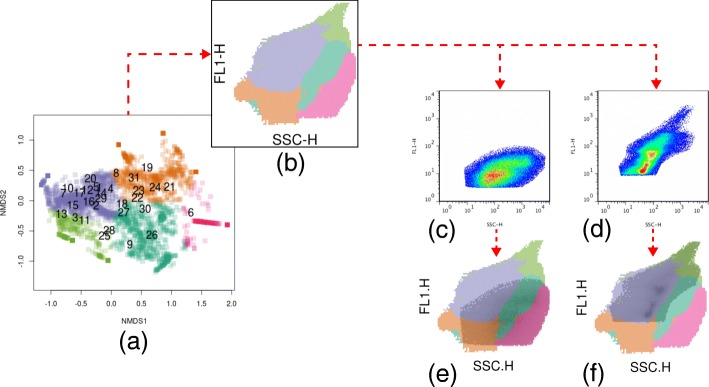
NMDS biplot **a** and mask of bins onto channels FL1-H and SSC-H **b**. Cytogram numbers 6 (**c**; Pond 7, S1) and 13 (**d**; Pond 13, S1) are overlaid by **b** to reveal how the known gated populations relate to ordination clusters (**e** and **f**). Dotted red arrows indicate the logical pathway through the figures

The biplot of the samples and bins, based on channels FL1-H and SSC-H, showed a broadly common area shared by most of the cytograms (blue and green clusters, Fig. 5a), as could be anticipated from the nestedness patterns from previous sections (Fig. 4b). Samples were differently associated with specific clusters of bins, which subsequent visual inspection revealed to correspond, partially or totally, to known cytometric subpopulations (Figs. 5c-f and Additional file 1: Figure S6)).

#### Pairwise comparisons

flowDiv and FlowFP were the only pipelines that significantly and positively correlated with DGGE information (Mantel statistic r = 0.20 and 0.19, respectively) Additional file 2: Figure S7. Those techniques were also highly correlated (Mantel statistic r = 0.65), probably due to their common principles (i.e., binning-based techniques) (Table 1).

**Table 1:** Mantel statistics based on Bray-Curtis distance matrix calculated for pairwise comparisons of pipelines

	DGGE	CHIC	Dalmation plot	CyBar	flowFP	PhenoFlow	flowDiv
DGGE	-						
CHIC	0.05	-					
Dalmation plot	-0.05	0.06	-				
CyBar	-0.07	-0.07	-0.11				
flowFP	0.18^∗^	0.13	-0.34	0.42^∗^	-		
PhenoFlow	0.10	0.08	-0.35	0.15	0.37^∗^	-	
flowDIV	0.20^∗^	0.12	-0.20	0.12	0.65^∗^	0.22^∗^	-

Asterisks (∗) represent significant results at *α*=0.05

Notably, these results are in line with previously published reports that described the correlation between molecular traits and cytometric diversity [16, 39].

Although flowDiv did not correlate significantly with the remaining techniques, the discrepancies could be interpreted merely as a matter of tuning, caused by differences in their default working principles [6, 16].

## Conclusions

The need to both reduce the analytical subjectivity and emphasize more practical aspects of environmental flow cytometry studies causes a paradigm shift so as to harmonize objectivity with applicability. flowDiv provides a fast, low-cost, straightforward, and rather intuitive way of proceeding with this kind of analysis, as it combines formal mathematical solutions and biological rationales in an intuitive framework specifically designed to explore cytometric diversity. In addition to solving some important technical issues, such as the perspective correction of differently acquired datasets, flowDiv provides an intelligible foundation for the use of multidimensional contingency tables in environmental FCM analyses. On the one hand, multidimensional contingency tables resolve quite efficiently for multicolor assays, since they maintain an epistemological relationship to the fairly well-known ecological tables. This property permits a more straightforward biological interpretation of diversity indices derived from FCM data. On the other hand, their summaries by biplots, along with a further clusterization and mapping of bins back to cytograms, constitute an elegant strategy to understand the global and local behaviors of FCM populations in the cytometric fingerprint.

flowDiv is a flexible and robust analytical method for considering FCM data analysis. We hope that it will be a useful tool for environmental and non-environmental cytometrists, since there are clearly many possible avenues for expanding its applications, from environmental monitoring to data-quality assessment of FCM experiments. As an open-source initiative we hope that flowDiv will be considered, studied and improved by cytometrists from all fields of expertise in which it may be useful, both environmental and others.

## Availability and requirements

**Project name:** flowDiv**Project home page:**https://cran.r-project.org/web/packages/flowDiv/**Operating system(s):** Platform independent**Programming language:** R**Other requirements:** R 2.16.0 or higher**License:** GPL-3**Any restrictions to use by non-academics:** no restrictions

## Additional files

Additional file 1 Cytograms and masks of bins overlaid onto channels FL1-H and SSC-H for all 31 Patagonian lakes used in this study. (PNG 11400 kb)

Additional file 2 Heatmaps based on distance matrices (Bray-Curtis distance) for the Patagonian lakes used in this study. Data are from: (a) DGGE, (b) CHIC, (c) flowCyBar, (d) Dalmation Plot, (e) FlowFP, (f) PhenoFlow, and (g) flowDiv pipelines. Dendrograms were based on Ward’s hierarchical agglomerative clustering method. (PNG 1810 kb)

## Abbreviations

ANOVA: Analysis of Variance
CHIC: Cytometric Histogram Image Comparison
Chla: Chlorophyll a
CyBar: Cytometric barcoding
DGGE: Denaturing Gradient Gel Electrophoresis
DOC: Dissolved Organic Carbon
DN: Dissolved Nitrogen
DO: Dissolved Oxygen
FCM: Flow Cytometry
*K*_*d*_: Diffuse Attenuation Coefficient
Lat: Latitude
Lon: Longitude
nMDS: Non-Metric Multidimensional Scaling
PCA: Principal Component Analysis
PCR-DGGE: Polymerase Chain Reaction-Denaturing Gradient Gel Electrophoresis
PERMANOVA: Permutational Multivariate Analysis of Variance
SSC: 90^∘^ Side Scatter
Temp: Temperature
