# Supplementary figures and images for: flowDiv: a new pipeline for analyzing flow cytometric diversity
Source: BMC Bioinformatics. 2019 May 28;20:274. doi: 10.1186/s12859-019-2787-4 (PMC6540361; doi:10.1186/s12859-019-2787-4)

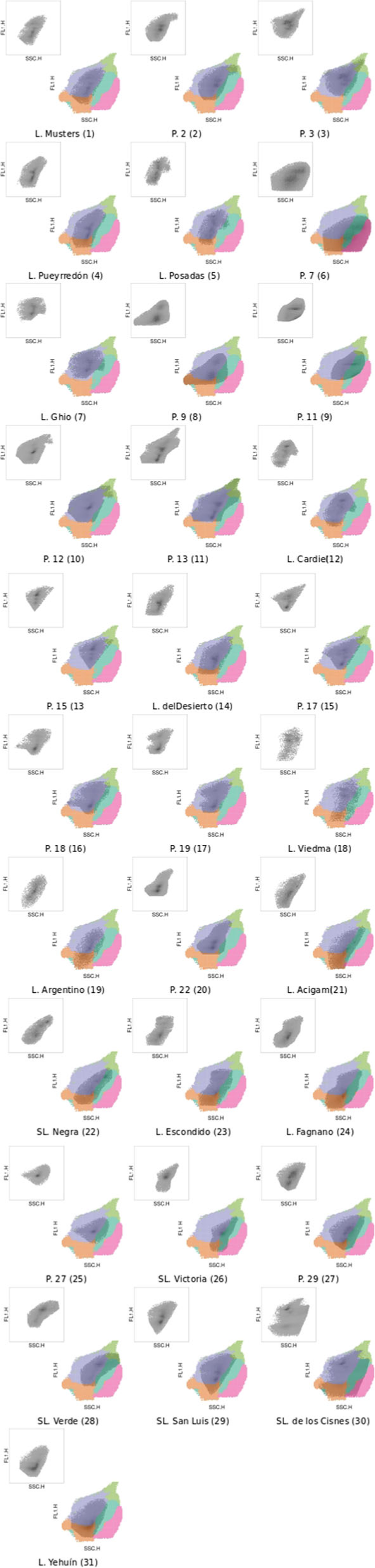

Supplement: Supplementary file 1 — Cytograms and masks of bins overlaid onto channels FL1-H and SSC-H for all 31 Patagonian lakes used in this study. (PNG 11400 kb) [file 12859_2019_2787_MOESM1_ESM.png]

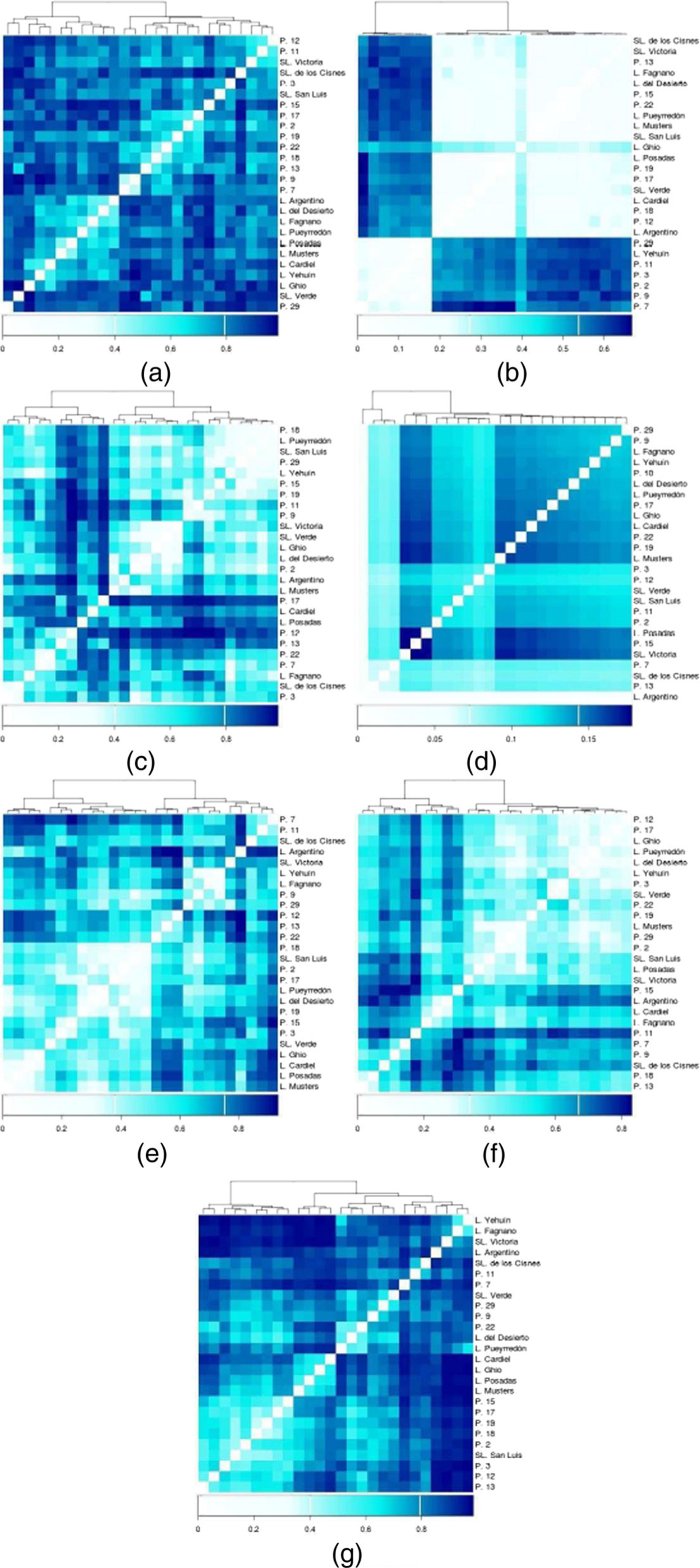

Supplement: Supplementary file 2 — Heatmaps based on distance matrices (Bray-Curtis distance) for the Patagonian lakes used in this study. Data are from: (a) DGGE, (b) CHIC, (c) flowCyBar, (d) Dalmation Plot, (e) FlowFP, (f) PhenoFlow, and (g) flowDiv pipelines. Dendrograms were based on Ward’s hierarchical agglomerative clustering method. (PNG 1810 kb) [file 12859_2019_2787_MOESM2_ESM.png]
